# Three-Dimensional Printing Parameter Assessment of Elastomers for Tendon Graft Applications

**DOI:** 10.3390/biomimetics10110785

**Published:** 2025-11-19

**Authors:** Trent Lau, Ashley Talwar, Bijan Abar, Samuel B. Adams

**Affiliations:** 1 Department of Orthopedic Surgery, Duke University School of Medicine, Durham, NC 27710, USA; trent.lau@duke.edu (T.L.);; 2 Department of Biomedical Engineering, Duke University Pratt School of Engineering, Durham, NC 27708, USA; 3 Department of Emergency Medicine, Duke University School of Medicine, Durham, NC 27710, USA

**Keywords:** 3D printing, additive manufacturing, elastomers, TPU, synthetic grafts, tendon grafts

## Abstract

Additive manufacturing has significantly advanced patient-specific medical devices, particularly for hard tissue repair, yet applications in soft tissue remain limited. Existing approaches for 3D-printed soft tissue devices employ mainly biogels and bioinks for regenerative purposes, while synthetic grafts for tendons and ligaments remain non-customizable in shape and mechanics. This study investigates the mechanical performance of 3D-printed thermoplastic polyurethane (TPU) elastomers as a function of printing parameters, informing customizable connective tissue graft designs. Type C dogbone specimens (*n* = 180) of three replicates each of parameter combinations from material shore hardness, presence of anchoring within the lattice, infill patterns, and infill density were printed and tested following modified ASTM D412 standards for vulcanized rubber and elastomers. The measured mechanical properties are elastic modulus, tensile yield stress, yield strain, ultimate tensile strength, and ultimate strain. Results show that shore hardness and infill density are the strongest predictors of mechanical properties, with positive but modest effects from anchor presence. Infill pattern is only significant through interactions, and its effects depend on other parameters. While all groups underperformed compared to manufacturer-reported TPU strengths and were well below in vitro tendon failure loads, findings highlight material selection and density optimization as critical early considerations for future patient-specific elastomeric graft design.

## 1. Introduction

Recent advances in additive manufacturing have allowed many new innovations in all fields of medicine ranging from patient-specific medical implants and instrumentation to regenerative medicine in the form of bioprinting [1]. In the field of orthopedic surgery, while there are a growing number of commercial applications of additive manufacturing for bony mass defects and reconstructions, there are much fewer offerings for soft tissue defects and reconstructions. The approach for soft tissue medical 3D printing has largely been centered on hydrogels and bioinks, which serve to facilitate tissue regeneration rather than acting as a mechanical replacement and generally leaves patients without mechanical function for long periods of time [2].

In orthopedic surgery, the current offerings for tendon reconstructions with mass deficits are limited to autografts, allografts, and synthetic grafts. The consequences of autografts are limited by donor-site morbidity, reduced mechanical strength, and the lack of intrinsic regenerative capacity, which underscore the need for more advanced tissue-engineered solutions [3]. The consequences of allografts are immunological rejection and possible disease transmission [4]. Synthetic graft products avoid the immunological risks of allografts and the donor site morbidities risks of autografts [4,5]. However, these grafts often require intraoperative preparation to match the patient’s anatomy, prolonging OR time and increasing the risk of infection, blood loss, and cost [5]. Furthermore, because commercially available synthetic grafts are usually made from a homogenous band of polymer, they are somewhat adaptable in shape from intraoperative trimming, but they are not customizable in intrinsic mechanical properties. This is currently an unaddressed need because the mechanical properties within a tendon changes depending on the location, such as how the properties in the midsubstance of a tendon are different from the properties near the insertion sites [6]. This mismatch in mechanical properties between the graft and surrounding tissue leads to a phenomenon known as stress shielding, in which the surrounding tissues degenerate over time and increase the risk of graft failure [7] due to uneven force distribution leading to biological degradation [8]. In fact, stress shielding can occur even when grafts are not used, as seen with non-absorbable suture augmentation [8]. As such, there is a need for minimizing the mismatch of force distribution and mechanical properties for all types of soft tissue repairs.

Therefore, what this study aims to do is to begin using FDM (fused deposition modeling) 3D-printing of TPU (thermoplastic urethane) to explore the relationship between certain 3D-printing parameters on the mechanical properties of elastomeric parts. While tendon function depends on a broad set of properties—including viscoelasticity, fatigue resistance, three-point bending, compression, and biological integration—this study focuses specifically on tensile properties. Tensile testing was selected as a first step because it represents the primary loading mode of tendons and provides standardized comparability through ASTM D412. This brings us a step closer to our goal of eventually creating patient-specific TPU tendons that can match patient anatomy perfectly and match viscoelastic properties of the native tendon based on location of the defect, offering an unprecedented level of adaptability for large tendon deficits. This would not only save operating room time while avoiding the consequences of autografts and allografts such as immunological reactions [9] but also have the potential to support cell integration into the implant and even allow reservoirs for additional additives like antibiotics or biologics for further treatment customizability by manufacturing unique and functional 3D-geometries achievable only with additive manufacturing.

## 2. Materials and Methods

The primary outcomes of this study are the elastic modulus, tensile yield stress, and ultimate tensile strength of 3D-printed TPU elastomers, using the ASTM D412 standard [10] testing procedure for vulcanized rubber and thermoplastic elastomers. As adherent to the standard, the ASTM D412 Type C Dogbone specimen was printed with different slicing parameters on a Bambu Lab X1C Carbon (Shenzhen, China). For each printing profile tested in this study, the 3D printer used its own micro-Lidar visualization function: a laser-based sensing system that measures the height and surface contours of printed layers in real time. This function was used to calibrate the flow rate such that all printing profiles were printed at an equal material output rate with uniform settings across each profile besides the settings being tested as independent variables of interest.

The materials used in this study were Siraya Tech (San Gabriel, CA, USA) TPU filament at 95a shore hardness and 85a shore hardness. These materials were selected for its compliance with ISO 10993-10, -5, and -23 [11,12,13] biocompatibility protocols, which most commercial TPU filaments are not compliant with [14]. All filaments were dried at 70 °C for a minimum of 24 h using a Creality Space Pi filament dryer (Shenzhen, China) prior to use for printing. Printing for all samples occurred at 240 °C with all line widths set at 0.4 mm. The samples were stored in a sealed box until testing, which was performed at room temperature (~22–24 °C) in a classroom environment within 1–2 days of printing. TPU’s sensitivity to temperature and humidity, thereby, did not pose an issue: TPU softens above ~60–80 °C, becomes brittle below its Tg of ~−30 °C. Relative humidity was not actively monitored nor measured in fabrication nor testing environment. However, TPU performance issues are generally observed when RH exceeds 40–45%, and best long-term stability is achieved when RH is maintained below 15% [15].

**Figure 1:**
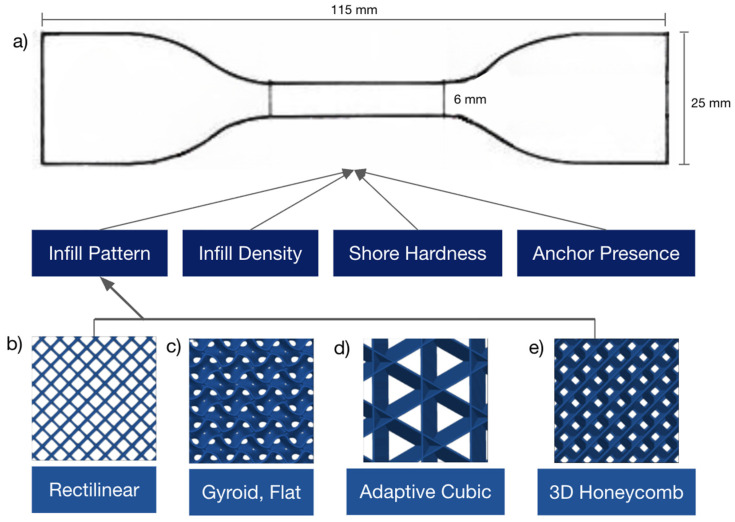
Three-dimensional printing parameters flow chart with bird’s eye view of the 20% infill pattern. (**a**) ASTM D412 Type C Dogbone specimen and flow chart of parameters modified. (**b**) Rectilinear. (**c**) Gyroid, Flat. (**d**) Adaptive Cubic. (**e**) 3D Honeycomb.

While it is known that perimeters of a 3D print contribute to tensile strength [16], the samples in this study are to explore how infill pattern and density affect tensile strength, and thus are printed without any perimeters, leaving the printed object’s geometric composition as an open cell. As for the infill anchor parameters, samples were printed with either zero infill anchors or the maximum available length as determined by the algorithm used by the slicing software of this study, Bambu Studio. Samples are printed in triplets across each infill density category ranging from 20%, 40%, 60%, 80%, and 99% for each infill pattern of interest, which, for this study, are gyroid flat, adaptive cubic, and 3D honeycomb (Figure 1). All samples were printed flat, meaning the largest sample surface was in the XY plane of the printing bed for ease of manufacturing.

**Figure 2:**
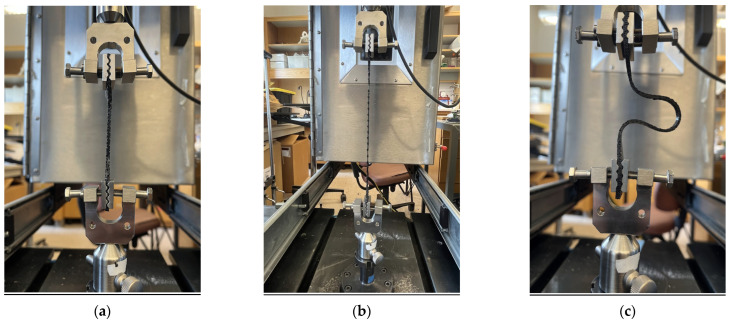
Sample loading and testing on a universal testing machine. (**a**) An example of a specimen loaded on a Universal Testing Machine and (**b**) tested under uniaxial tensile load, (**c**) demonstrating plastic deformation after exceeding the yield stress. The specimen did not fracture and could not reform to its original shape.

After samples were printed, in accordance with ASTM D412 procedures, the cross-sectional area of each sample’s gauge was measured. Then, they were properly aligned using a jig and placed in a TestResources 830LE-AT with a FAT10K-6K Axial Torsion 1000 N Load Cell with wave grips (Shakopee, MN, USA). The displacement rate was set to 500 mm/min until failure, which was defined as total breakage across the entire gauge cross-sectional area or the maximal travel distance of the testing machine was reached (Figure 2).

Load (N) and time (s) data were recorded and converted to stress and strain values. Stress was calculated by dividing the load by the measured cross-sectional area, which was determined using the formula: area = thickness × width. Infill density was not incorporated into the cross-sectional area calculation, as doing so would not meaningfully correct for variations in density. The ASTM standard defines stress based on the nominal geometry of the specimen rather than the effective material distribution, and incorporating infill into the area term would obscure the actual influence of internal structure on performance. Instead, infill density was treated as an independent variable that directly affects mechanical behavior through changes in internal porosity and mass distribution. In other words, all stress calculations were based on the measured external geometry of the gauge section to maintain comparability while allowing infill density to emerge as the key design parameter influencing tensile properties.

**Figure 3:**
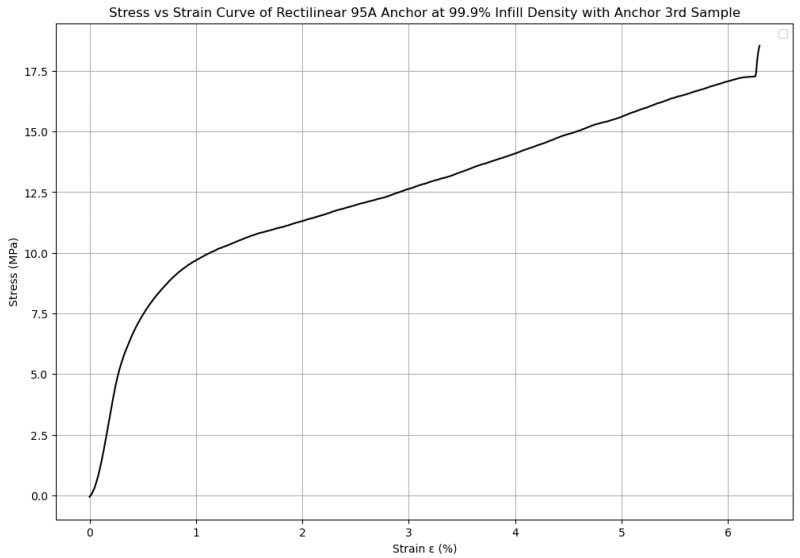
Savitzky–Golay-filtered ASTM D412 stress–strain curve of Sample #3 (Rectilinear 95A dogbone printed at 99.9% infill density with anchor). The processed curve highlights the primary elastic and plastic deformation behavior up until the ultimate tensile strength value (UTS). Samples had failed at varied points either at or past the UTS; therefore, the plots were standardized to the ultimate tensile strength value.

Strain was calculated from displacement, which in turn was derived from the time and crosshead speed using the formula: displacement = (time × crosshead speed)/60; strain = displacement/gauge length [10]. To reduce noise in the stress vs. strain data while preserving local features, a Savitzky–Golay filter was applied prior to further analysis [17] and an example is shown in Figure 3 below.

Mechanical properties were extracted from the filtered stress–strain curves. Young’s modulus of elasticity was determined by performing linear regression on a 15-point window within the initial linear elastic region that produced the maximum slope, representing the elastic modulus.

Yield stress and yield strain were identified using the 0.2% offset method [18], wherein a line offset by a strain of 0.002 was drawn parallel to the elastic slope, and its intersection with the curve was used to define the onset of plastic deformation. Ultimate tensile strength and ultimate strain were defined as the peak stress and its corresponding strain value before failure. As a note, ultimate tensile stress and ultimate tensile strength are used interchangeably.

### Statistical Analysis

To evaluate the effects of experimental conditions on mechanical properties, a comprehensive set of statistical analyses was conducted, using the data set containing the infill patterns gyroid flat, 3D honeycomb, and adaptive cubic only (no rectilinear). Prior to inferential testing, Levene’s test for equality of variances was used to assess the assumption of homogeneity of variances across condition groups for each dependent mechanical outcome: modulus, yield stress, and ultimate strength. The results of Levene’s test were used to validate assumptions for subsequent parametric procedures and confirmed the suitability of variance-based methods for group comparisons.

One-way analyses of variance (ANOVAs) were conducted to examine the main effects of the categorical independent variables—pattern type, infill density, anchor type, and shore hardness—on each mechanical property. To evaluate interaction effects among independent variables, factorial ANOVAs were performed, incorporating two-way and higher-order interaction terms.

To control for continuous variation in structural design, analysis of covariance (ANCOVA) models were implemented. Infill density, although initially a factor, was treated as a continuous numeric covariate in these models to better reflect its scalar nature and improve model sensitivity. This approach allowed for the estimation of adjusted group means while accounting for the linear influence of infill density on mechanical outcomes. The ANCOVA and univariate regression outputs are reported in the Supplementary Materials.

For all univariate regression analyses, model assumptions of normality and homoscedasticity were rigorously assessed. Residuals were assessed for normality via Q-Q plots and the Shapiro–Wilk test, while homoscedasticity was examined through residuals-versus-fitted plots. Although residual variance was consistent across levels of the predictors, the distribution of residuals deviated from normality. The clustered patterns observed in residual plots suggested that this non-normality was attributable to the use of binary or categorical predictors in the univariate models, rather than to unequal error variance or poor model fit. In light of this, multivariate techniques were used to more accurately capture the complex relationships among variables and account for the intercorrelations between outcomes.

To examine the joint influence of each independent variable across all mechanical outcomes, multivariate analysis of variance (MANOVA) was conducted. Wilks’ Lambda was used as the primary test statistic due to homogeneity of variances by the Levene’s test and equal sample sizes per condition, and the data was further supplemented by Pillai’s Trace, Hotelling–Lawley Trace, and Roy’s Greatest Root. To complement these tests, multivariate regression was also used to model the continuous relationships between predictors and multiple mechanical outcomes. Both MANOVA and multivariate regression analyses were conducted twice: once without interactions and with interactions in order to evaluate their potential influence on the overall outcomes. The reference condition was defined as the combination of no anchor, 3D honeycomb pattern, 85A filament, and 20% infill density. This group consistently exhibited the lowest values across all dependent variables and was thus used as the baseline in all models for comparative interpretation.

## 3. Results

Table 1 presents the measured values of elastic modulus, yield stress, and ultimate tensile strength for samples printed with 99.9% infill density and shore hardness 95A, comparing configurations with and without anchoring across four pattern types. In all patterns, the inclusion of an anchor led to consistently higher mechanical property values. The Rectilinear pattern exhibited the highest elastic modulus values in both configurations (24.78 ± 0.71 MPa with anchor; 24.08 ± 0.17 MPa without), indicating superior stiffness. Three-dimensional Honeycomb also showed strong performance, though anchoring notably increased its ultimate yield (16.83 ± 0.27 MPa vs. 14.64 ± 0.10 MPa). The adaptive cubic pattern produced the lowest overall values across all metrics, particularly in the absence of anchoring. The gyroid flat pattern, while less stiff than Rectilinear, demonstrated competitive tensile and ultimate strengths, especially when anchored. Across all patterns, the anchor contributed more prominently to enhancements in tensile and ultimate properties than to elastic modulus, highlighting its role in load transfer and failure resistance under tensile conditions.

**Figure 4:**
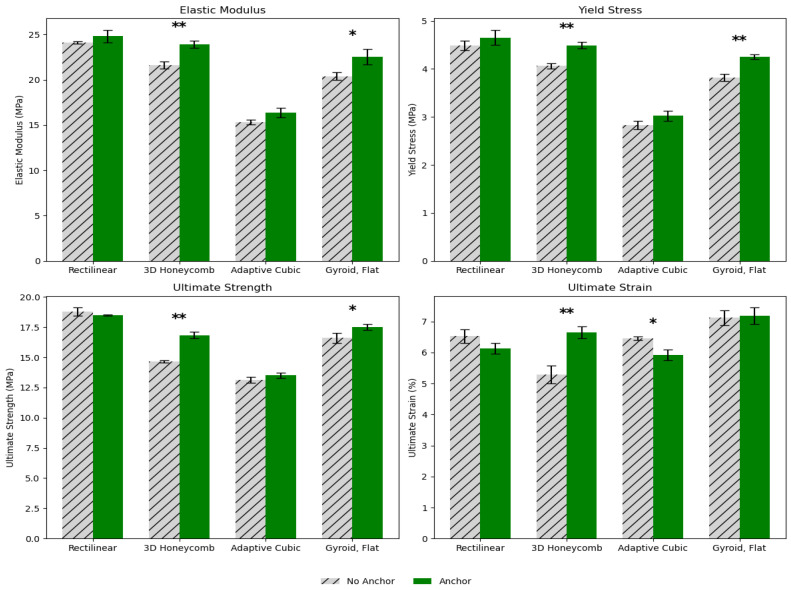
Comparison of anchor vs. no anchor across infill patterns. All samples were printed with 99.9% infill and 95A shore hardness. Error bars represent standard deviation. Statistical significance is denoted as *p* < 0.05 (*), *p* < 0.01 (**).

**Figure 5:**
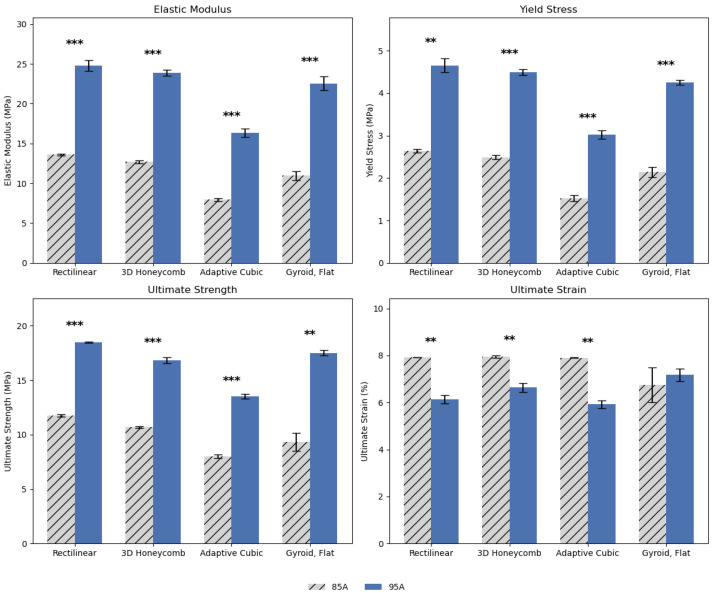
Comparison of shore hardness between 85A and 95A across infill patterns. All samples were printed with 99.9% infill and included anchors. Error bars represent standard deviation. Statistical significance is denoted as *p* < 0.01 (**), *p* < 0.001 (***).

Figure 4 and Figure 5 illustrate the combined effects of anchoring and shore hardness on key mechanical properties across four infill patterns, with all samples printed at 99.9% infill. Anchoring consistently enhanced performance across nearly all metrics, with anchored specimens exhibiting higher elastic modulus, yield stress, and ultimate tensile strength compared to non-anchored samples, particularly in Rectilinear and 3D Honeycomb patterns, where stiffness and strength improvements were most pronounced (*p* < 0.05 to *p* < 0.001). While gains in ultimate strain were more pattern-dependent, anchoring generally improved overall mechanical resilience. Furthermore, materials with shore hardness 95A consistently outperformed those with 85A across all metrics and infill patterns, indicating increased stiffness, strength, and in some cases, improved ductility. Statistically significant differences were observed in nearly all comparisons, with *p*-values < 0.001, particularly in elastic modulus and ultimate strength, where 95A samples demonstrated substantial gains. For example, the rectilinear and gyroid flat patterns showed large increases in modulus and ultimate strength with the harder material. While ultimate strain generally decreased with increasing hardness (indicating reduced elongation), this effect was pattern-dependent and less pronounced in gyroid flat. These results suggest that both anchoring and increasing shore hardness enhances mechanical performance, especially stiffness and strength, though potentially at the expense of ductility in certain geometries.

**Figure 6:**
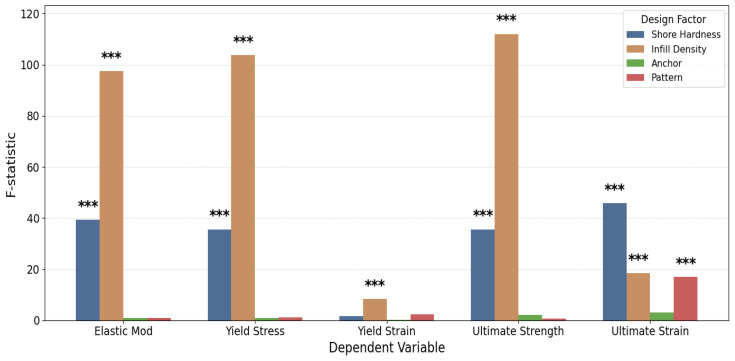
Main effects by F-statistics from one-way Type II ANOVA models. Statistical significance is denoted as *p* < 0.001 (***).

One-way ANOVA analyses demonstrated distinct differences in the relative influence of the four design factors on TPU mechanical behavior. Infill density emerged as the most dominant determinant across all strength- and stiffness-related outcomes, showing the largest F-statistics for elastic modulus, yield stress, and ultimate tensile strength. Shore hardness was the next most influential factor, exerting substantial effects on modulus, yield stress, and strain-related responses. In contrast, anchor presence exhibited minimal influence, with only weak or nonsignificant contributions to mechanical performance. Pattern effects were generally small and nonsignificant, except for ultimate strain, where it showed a notable main effect (Figure 6). Overall, these findings identify infill density and shore hardness as the primary drivers of TPU mechanical properties, while anchor and pattern play minor, outcome-specific roles.

**Figure 7:**
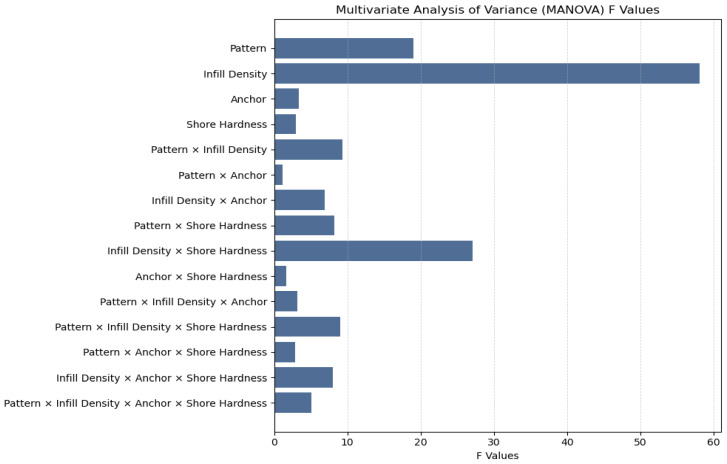
*F*-Statistics for main and interaction effects in MANOVA using Wilk’s Lambda. All main effects and nearly all interactions were statistically significant, except for pattern × anchor and anchor × shore hardness.

Extending this analysis, a multivariate analysis of variance (MANOVA) was performed to assess the significance of both main effects and higher-order interactions among the design factors—pattern, infill density, anchor presence, and shore hardness—on mechanical performance outcomes. Using Wilks’ lambda *F* statistics (Figure 7), infill density produced the largest main effect (*F* = 58.10), indicating it is the single strongest driver of overall mechanical response. Pattern was the next most influential main effect (*F* = 19.04). Anchor presence (*F* = 3.39) and shore hardness (*F* = 3.00) produced smaller but statistically significant main effects. For two-way interactions, the largest effect was infill density × shore hardness (*F* = 27.09), showing a notable dependence of mechanical behavior on the combination of internal structure and material stiffness. Other significant two-way interactions included pattern × infill density (*F* = 9.33), infill density × anchor (*F* = 6.87), and pattern × shore hardness (*F* = 8.19). By contrast, pattern × anchor (*F* = 1.12) and anchor × shore hardness (*F* = 1.64) were not significant. Among three-way interactions, pattern × infill density × shore hardness (*F* = 9.05) and infill density × anchor × shore hardness (*F* = 8.02) showed moderate effects, while pattern × infill density × anchor was small but significant (*F* = 3.20). The four-way interaction was modest (*F* = 5.08). Collectively, these Wilks’ lambda results emphasize that infill density and its interactions—particularly with shore hardness—are the dominant drivers of mechanical behavior, while other parameters and many higher-order combinations have smaller or outcome-specific impacts.

**Figure 8:**
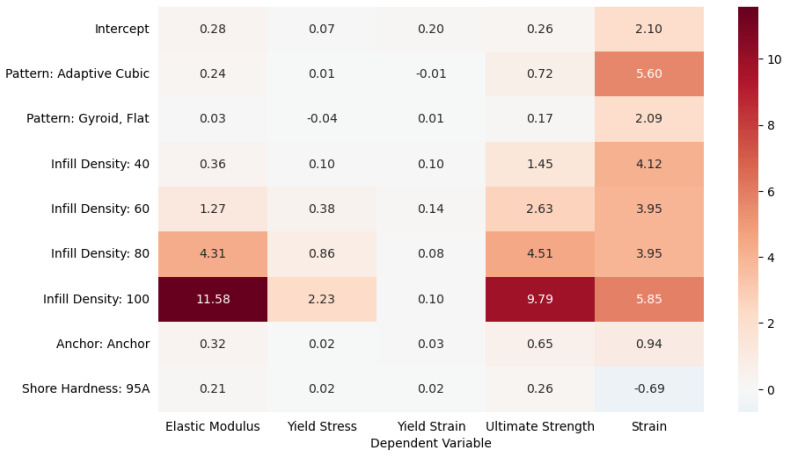
Heat Map of Multivariate Regression Coefficient (Main Effects from Interactions Data).

Multivariate linear regression was performed to quantify the main effects of individual manufacturing parameters on key mechanical responses, as shown in Figure 8. Across all dependent variables, infill density emerged as the dominant predictor of mechanical performance, with coefficients increasing steadily across density levels. The fully dense condition (100%) produced the strongest effects on elastic modulus (β = 11.58), ultimate strength (β = 9.79), and Strain (β = 5.85), reflecting pronounced gains in stiffness, strength, and ductility at maximum infill. Intermediate densities (60–80%) contributed moderate positive effects, while low infill (40%) produced comparatively small improvements. Pattern type also influenced performance, though to a lesser extent. The adaptive cubic pattern consistently exhibited slightly higher coefficients than gyroid, flat, especially for ultimate strength (β = 0.72 vs. 0.17) and Strain (β = 5.60 vs. 2.09), indicating modest but measurable gains in both stiffness and deformability. Anchor presence exerted a mild positive influence, increasing elastic modulus (β = 0.32) and ultimate strength (β = 0.65), though its effects on yield stress and strain were minimal. In contrast, shore hardness had a limited overall effect: switching from 85A to 95A filament yielded small positive shifts in elastic modulus (β = 0.21) and ultimate strength (β = 0.26), but negligible or slightly negative effects on strain-based metrics (β ≈ −0.69). These results confirm that infill density is the primary driver of mechanical performance in multivariate models, with secondary contributions from pattern geometry and anchoring. Filament Hardness, while directionally consistent with stiffer responses, played a comparatively minor role.

### Results Summary

To evaluate the mechanical performance of 3D-printed TPU specimens, a comprehensive suite of statistical models was applied across five key outcome variables: elastic modulus, yield stress (σ), yield strain, ultimate strength (σ), and Total Strain. The design factors examined included pattern, infill density, anchor, and shore hardness. All regressions used the baseline parameter combination of Honeycomb as the infill pattern, 20% for infill density, no anchor for anchor presence, and 85A for shore hardness.

The main effects and interactions were assessed using ANOVA and MANOVA, and predictive modeling was further performed using additional analyses, including univariate regression, multiple linear regression and ANCOVA (see Supplementary Figure S1, Tables S1 and S2, respectively), were consistent with these findings and are provided in the Supplementary Materials.

Across all statistical frameworks, infill density consistently emerged as the dominant predictor of mechanical behavior. As shown in the one-way ANOVA results (Figure 6), infill density produced the highest F-statistics across all stiffness- and strength-related outcomes, confirming its primary influence on elastic modulus, yield stress, and ultimate strength. This trend persisted in the multivariate analyses, where both MANOVA (Figure 7) and multivariate linear regression (Figure 6) demonstrated that coefficients increased systematically with density level, highlighting the pervasive role of density in enhancing both stiffness and ductility. In the ANCOVA models (see Supplementary Table S2), where infill density was treated as a covariate, it remained the most significant predictor across all mechanical responses, exhibiting the largest F-values and maintaining strong predictive power even in the presence of higher-order interaction terms. Higher densities consistently yielded superior mechanical performance, particularly in elastic modulus and ultimate strength, where fully dense (99.9%) specimens demonstrated substantial improvements relative to lower-density counterparts. Together, these results establish infill density as the most critical design factor governing the stiffness and strength of printed TPU components, exerting a robust and positive influence across all statistical modeling approaches.

Shore hardness emerged as the second most influential factor after infill density, maintaining a strong and consistent positive influence across nearly all mechanical responses. As illustrated in Figure 4 and Figure 5, increasing shore hardness from 85A to 95A produced substantial gains in stiffness and strength across all infill patterns and densities, confirming the material’s enhanced load-bearing capability. This trend remained robust in the ANCOVA results (see Supplementary Table S2), where shore hardness exhibited some of the highest F-values among main effects, second only to infill density. The harder filament significantly elevated elastic modulus, yield stress, and ultimate strength, underscoring its central role in improving overall structural performance. Furthermore, shore hardness interacted strongly with infill density, producing pronounced synergistic effects that amplified stiffness and strength at higher densities. Collectively, infill density and shore hardness accounted for the majority of the explained variance in mechanical outcomes, establishing them as the two dominant parameters governing the stiffness and strength of printed TPU specimens.

As for anchor presence, the use of perimeter anchoring alone did not exhibit statistically significant main effects across any of the measured outcomes in the one-way ANOVA (Figure 6), indicating that anchoring by itself did not substantially alter elastic modulus, yield stress, yield strain, or ultimate strength under isolated testing conditions. However, multivariate and factorial analyses revealed that its contribution becomes more pronounced when considered in combination with other design factors. As shown in the MANOVA (Figure 7) and confirmed by ANCOVA (see Supplementary Table S2), anchor presence participated in several significant interaction terms—most notably with infill density and shore hardness—suggesting that its effect depends strongly on the material and structural context. These interactions highlight that anchoring can enhance load transfer and delay failure initiation, particularly in denser and stiffer configurations. Although anchor presence remained insignificant for yield strain and ultimate strain when examined as an isolated main effect, higher-order terms demonstrated that it can influence strain capacity under specific geometric or material combinations. The multivariate regression results (Figure 8) further illustrate this conditional behavior: anchoring modestly increased elastic modulus (β = 0.32) and ultimate strength (β = 0.65) but had minimal influence on strain-based properties. Among pattern types, 3D Honeycomb exhibited the largest relative improvements with anchoring, gyroid flat showed moderate gains, and adaptive cubic demonstrated smaller yet inconsistent increases in stiffness and strength. Overall, while anchor presence was not a dominant main factor, it acted as an important modifier that improved performance primarily through its synergistic interactions with geometry, density, and material stiffness.

Infill pattern, when evaluated as an isolated main effect, was generally not a significant predictor of mechanical performance, as indicated by the one-way ANOVA results (Figure 6) and univariate regressions (Figure S1). Among the measured outcomes, only ultimate strain exhibited a notable main effect attributable to pattern geometry, while elastic modulus, yield stress, and ultimate strength remained largely unaffected when pattern was analyzed independently. However, when controlling for additional design factors or including interaction terms, pattern effects became more prominent. Both the MANOVA (Figure 7) and ANCOVA/regression analyses (Figure 6 and Figure 7, Supplementary Table S2) demonstrated that infill pattern gained statistical significance when combined with infill density and shore hardness, underscoring its contextual influence on mechanical response. The interaction between pattern and density exerted a particularly strong effect, especially at higher shore hardness (95A), where geometric architecture modulated stiffness and strength outcomes. Under these conditions, the 3D honeycomb consistently outperformed adaptive cubic and gyroid flat pattern across elastic modulus, and yield stress. This advantage became more pronounced at moderate-to-high infill levels (≥60%), where gyroid flat’s continuous, smooth internal surfaces likely facilitated more uniform stress distribution. At 80% and near-fully dense (99.9%) infill, this performance gap further widened, particularly in ultimate strength, while under Shore 85A, pattern-related effects were smaller and less consistent. Comparisons with 3D honeycomb under softer material conditions (85A) yielded no statistically significant differences across densities, suggesting that pattern geometry plays a greater role in stiffer materials and denser structures. Overall, infill pattern emerged as a secondary but context-dependent determinant of TPU performance—exerting minimal influence in isolation yet contributing meaningfully through its interactions with density and material hardness to shape the balance between stiffness, strength, and ductility.

Collectively, the results from all statistical frameworks demonstrate a high degree of consistency and confirm that the selected printing parameters— infill density, shore hardness, Anchor Presence, and infill pattern—explain a substantial portion of the variability in the mechanical behavior of printed TPU specimens. Multiple linear regression analyses (see Supplementary Table S1) focused on the main effects revealed that these parameters accounted for nearly all variance in stiffness- and strength-related properties (R^2^ = 0.887 for elastic modulus, 0.887 for yield stress, and 0.904 for ultimate strength), while strain-based outcomes were less predictable (R^2^ = 0.196 for yield strain and 0.679 for ultimate strain). When interaction terms were included, explanatory power increased dramatically across all mechanical responses, achieving near-complete model fit (R^2^ = 0.999 for elastic modulus, 0.998 for yield stress, 0.998 for ultimate strength, 0.425 for yield strain, and 0.941 for ultimate strain). These results highlight that the mechanical performance of TPU is governed not solely by individual parameters but by their synergistic interactions, particularly between infill density and shore hardness, and, to a lesser extent, their coupling with anchor presence and pattern geometry. Collectively, these factors and their interactions underpin the observed trends across all analyses—showing that increasing density, material stiffness, and geometric reinforcement consistently enhances elastic modulus, yield stress, and ultimate strength, while their combined effects also modulate strain behavior in a pattern- and context-dependent manner.

## 4. Discussion

Our results indicate that for the purposes of creating synthetic grafts for connective tissue repairs, the most important variables are material selection (represented here as infill density and shore hardness) as indicated by their significance alone and in combination with each other (Figure 4 and Figure 5). Anchor presence has the least significant effects out of all four of the variables or is the least impactful when it comes to designing 3D-printed connective tissue grafts. Moreover, it is difficult to establish a definitive or universal order of impact across all analyses: infill density remains consistently dominant in both ANOVAs and MANOVA, but factors such as Pattern and shore hardness appear with varying levels of influence depending on whether interactions and multivariate dependencies are considered. Infill pattern should be selected based on mechanical needs, non-mechanical needs, and what other prior printing parameters.

Regression analysis further elucidates that for the three infill patterns tested, overall, adaptive cubic provides mechanical benefits in ultimate tensile strength, but these become statistically and practically meaningful only at 40% infill or greater with Shore 95A, and 80% or greater with Shore 85A, emphasizing the importance of higher infill density and increased shore hardness in unlocking pattern-driven performance gains.

And when considering all variable interactions, and at all levels of infill density, the patterns gyroid, flat, and 3D honeycomb appear to perform similarly. It is important to note that there are other outcomes predicted by infill pattern that are not addressed in this study, particularly under different loading conditions. Honeycomb and gyroid patterns can enhance compressive strength and energy absorption [19], whereas rectilinear and grid-like architectures may improve fatigue resistance under cyclic loading [20,21]. In addition, highly porous patterns such as gyroid flat facilitate cellular integration and tissue ingrowth [22]. As such, infill pattern should not be discounted in the design of synthetic grafts on account of this study.

Ultimately, our results are consistent with our expectations because the mechanical properties in a stress–strain curve for a material are inherent properties of the material itself at a molecular level [23] of which one aspect is represented here by infill density and shore hardness. However performance in mechanical testing can be influenced by geometry and anisotropy [19,20,21], which are aspects represented through infill density and infill pattern should be used to fine-tune mechanical needs, with anchor presence generally added for increased strength. For example, even when accounting for independent variable interactions, infill pattern largely remains minimally influential for most of the dependent variables of interest compared to the two other independent variables even if it is statistically significant. So, unless one has specific need cases like increasing ultimate strain or requiring high density prints for weight matching purposes, infill pattern should be selected based on other aspects untested in this study such as printing speed, scaffolding ability for cellular integration, or other mechanical properties not tested in this study like compression.

Notably, yield strain was the least responsive dependent variable to our selected predictors. Only infill density was statistically significant. This could suggest that strain at yield is less sensitive to the design factors of this study, or there is greater data variability is higher in this outcome, which can be due to mechanical or material characteristics unaccounted for in this study. Yield strain is believed to reflect the onset of polymer necking [24] in the struts, which appears to be more influenced by filament material and local cell geometry more than the other parameters like anchor presence.

The method of stress calculation for each sample highlights the importance of considering material structure in interpreting results. The ASTM D412 standard assumes a homogeneous, isotropic, and continuous material for all samples; however, in this study, the variation in infill density was not incorporated into the stress calculation and a modified standard was used. This assumption effectively treats the sample as a solid body, leading to an overestimation of the effective cross-sectional area that actually carries the load. At lower infill densities, the applied load is transmitted through fewer continuous material paths, resulting in higher localized stress concentrations and greater overall deformation. In contrast, higher infill densities provide more continuous load-bearing structures, enabling more uniform stress distribution and reduced strain under equivalent loading conditions. As a result, the nominal stress values may misrepresent the true internal stress distribution yet it has been elucidated that the relationship between infill density and mechanical strength is nonlinear, validating our approach [25]. The Gibson–Ashby model may be employed as a follow-up analysis to quantify this relationship, as it describes the dependence of elastic modulus and strength on the relative density of cellular materials through a power-law relationship, thereby providing a more accurate framework for interpreting the mechanical behavior of additively manufactured structures [26].

For the purpose creating a design paradigm for custom 3D printed Achilles tendon grafts, out of the dependent variables tested here, yield stress (the amount of stress a material can take before permanent deformation) was of the greatest interest because in a synthetic graft, permanent deformation at the elastic limit would be considered a failure of the implant, which would occur before breakage at the material’s ultimate tensile strength. The elastic modulus is also crucial because the inherent property of the material would determine how well the synthetic implant matches the elasticity of the surrounding tissue it is supposed to integrate with, which is a determinant of both performance and stress shielding. However, with these primary variables of interest in mind, it should be noted that all TPU samples printed with the parameter combinations reflected in this study fail to match reported mechanical properties of the Achilles tendon reported in other studies. It is notable that even the best performing combination of parameters for each dependent variable (Rectilinear, 95A, 99.9% infill density, Anchor Present), fails to mimic published data for fresh harvested human Achilles Tendon using tensile testing (see Supplementary Tables S11 and S12) [27] or Achilles tendon mechanical properties measured through other means [28,29,30].

Comparison with published Achilles tendon data [27] must be interpreted cautiously given differences in testing conditions and specimen properties. Human tendon samples are hydrated, viscoelastic, and subject to biological variability such as age-related crosslinking, whereas TPU samples are dry, homogeneous, and isotropic. In addition, Louis-Ugbo et al. tested at a displacement rate of 800 mm/min compared to our 500 mm/min, and tendon behavior is known to be strain-rate dependent. Cross-sectional geometry and gripping also differ substantially between irregular tendon tissue and uniform TPU dogbones [31]. Further, while TPU underperformed in absolute strength, its elastic region extended up to ~19% strain, compared to the Achilles tendon’s physiological elastic limit of only ~2% strain before plastic deformation. This suggests TPU grafts could remain elastic across the expected physiological loading range, thereby reducing risk of permanent deformation [32].

However, it is important to note that there are likely other confounding variables in the data collection of the Achilles tendon mechanical properties that are referenced in this study, such as differences or inconsistencies in cross-sectional area, varying crosslinkage of collagen in different portions of the tendon, cellular health of the samples, and differences in data collection methodology. Therefore, this study cannot fairly compare the mechanical property findings of 3D printed TPU to native human tendon and indicates further need to evaluate both native tendon mechanical properties and additional 3D printing parameters and combinations of parameters for 3D-printing elastomers in order to better elucidate the best way to adjust printing parameters to best match a desired tendon or ligament. Future experiments also include the mimicry of internal viscoelastic variation in human tendons (midsubstance vs. proximal aspect vs. distal aspect) by using subregional infill properties and parameter gradients within a single object, testing of a wider selection of other viscoelastic materials like silicone and other polyurethane based elastomers, nanoparticle-enhanced polymer composites, etc.

Additionally, it is important to acknowledge that the present study assessed only tensile properties, whereas tendon performance in vivo is determined by additional mechanical and biological characteristics. Fatigue resistance under cyclic loading for durability and resistance to repeated stress, viscoelastic time-dependent behavior, 3-point bending, and compressive responses at insertion sites are examples of future mechanical testing that can be conducted to more comprehensively evaluate graft longevity. Moreover beyond our studies’ findings of future experiments with shore hardness and infill density, additional 3D printing parameters may further enhance graft performance. For example, adjusting the cooling rate during deposition can improve interlayer adhesion and reduce void formation; annealing can increase crystallinity and stiffness in TPU [33]; and multi-material printing may enable the combination of elastomers with stiffer polymers or composites to achieve tendon-like mechanical properties. These strategies, in combination with optimized design parameters, represent promising future directions toward clinically viable tendon grafts.

## Figures and Tables

**Table 1:** Elastic modulus, yield stress, and ultimate yield of select samples. These samples had an infill density of 99.9% and shore hardness of 95A. The ± value is derived using the standard deviation of each respective measurement’s three trials.

	Elastic Modulus—Anchor	Elastic Modulus—No Anchor	Yield Stress—Anchor	Yield Stress—No Anchor	Ultimate Strength—Anchor	Ultimate Strength—No Anchor
Rectilinear	24.78 ± 0.71	24.09 ± 0.17	4.66 ± 0.16	4.51 ± 0.08	18.50 ± 0.05	18.78 ± 0.33
Three-dimensional Honeycomb	23.89 ± 0.38	21.63 ± 0.38	4.49 ± 0.07	4.07 ± 0.07	16.83 ± 0.27	14.64 ± 0.10
Adaptive Cubic	16.33 ± 0.54	15.31 ± 0.27	3.03 ± 0.10	2.83 ± 0.09	13.51 ± 0.21	13.13 ± 0.25
Gyroid, Flat	22.66 ± 0.61	20.37 ± 0.40	4.21 ± 0.14	3.83 ± 0.08	17.51 ± 0.25	16.60 ± 0.43

## Data Availability

The data presented in this study are available on request from the corresponding author due to the large size of the dataset.

## Supplementary Materials

The following supporting information can be downloaded at https://www.mdpi.com/article/10.3390/biomimetics10110785/s1: Figure S1: Univariate Regression Analysis of Mechanical Properties by Design Factor; Table S1: R2 values for each mechanical property under multiple linear regression, with and without interaction terms; Table S2: ANCOVA With Interaction Factors for Elastic Modulus, Yield Stress, and Ultimate Strength; Table S3: Adaptive Cubic TPU 85A; Table S4: Adaptive Cubic TPU 95A; Table S5: 3D Honeycomb TPU 85A; Table S6: 3D Honeycomb TPU 95A; Table S7: Gyroid Flat TPU 85A; Table S8: Gyroid Flat TPU 95A; Table S9: Rectilinear TPU 85A; Table S10: Rectilinear TPU 95A; Table S11: Mechanical Properties of fresh human Achilles Tendon by Louis-Ugbo et al; Table S12: Quantitative Comparison: Best TPU Sample vs. Human Achilles Tendon by Louis-Ugbo et al.

## Abbreviations

The following abbreviations are used in this manuscript:

UTS	Ultimate tensile strength
ANOVA	Analysis of Variance
MANOVA	Multivariate Analysis of Variance
ANCOVA	Analysis of Covariance
