# Supplementary material for: Three-Dimensional Printing Parameter Assessment of Elastomers for Tendon Graft Applications
Source: Biomimetics (Basel). 2025 Nov 19;10(11):785. doi: 10.3390/biomimetics10110785 (PMC12649998; doi:10.3390/biomimetics10110785)
Supplement: Supplementary file 1 [file biomimetics-10-00785-s001.zip › biomimetics-3879917-supplementary.pdf]

Supplementary Materials:

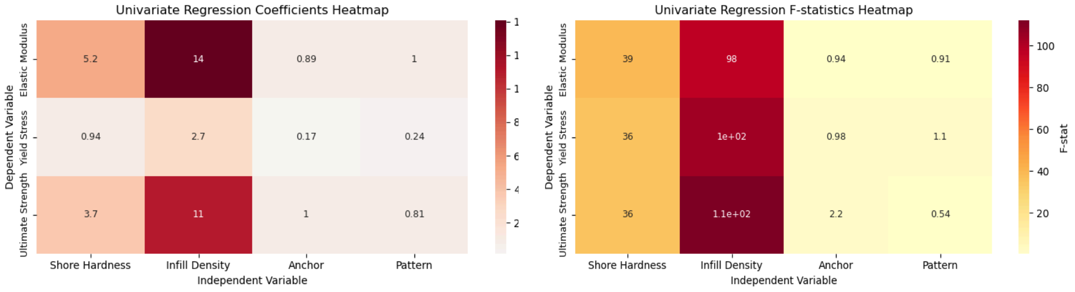

(a) **(b)**

Figure S1. Univariate Regression Analysis of Mechanical Properties by Design Factor. (a) Standardized regression coefficients and (b) F-statistics for shore hardness, infill density, anchor presence, and pattern design across elastic modulus, yield stress, and ultimate tensile strength. Infill density exhibited the strongest positive effects across all responses (β up to 14; $F \approx 98$–110), confirming its dominant influence on stiffness and strength. Shore hardness also contributed significantly (β up to 5.2; $F \approx 36$–39), while anchor presence and pattern design showed minimal or negligible effects ($|\beta| < 1.1$; $F < 2.3$). Overall, infill density and shore hardness were the primary determinants of mechanical performance, with anchor and pattern acting as weak secondary factors.

Table S1: R^2 values for each mechanical property under multiple linear regression, with and without interaction terms. Benjamini–Hochberg FDR correction was applied to regression with interaction terms. Printing parameters explain the majority of variance in strength outcomes (elastic modulus, yield stress, ultimate strength; $R^2 \geq 0.887$), and including interaction terms nearly fully explains variance in these strength measures ($R^2 \approx 0.998$ -- 0.999). Strain responses—particularly yield strain—were less predictable from main effects alone ($R^2 \approx 0.196$) but showed notable improvement when interaction terms were included ($R^2 \approx 0.425$ for yield strain; $R^2 \approx 0.941$ for ultimate strain). Overall model F-tests for both main-effects and interaction models were highly significant (all overall $p \ll 0.001$), and Benjamini–Hochberg FDR correction was applied to control false discovery when interpreting coefficients from the interaction models.

**Dependent Variable**	**R^2 (Main Effects Only)**	**R^2 (With Interactions)**
Elastic Modulus	0.887	0.999
Yield Stress	0.887	0.998
Ultimate Strength	0.904	0.998
Yield Strain	0.196	0.425
Ultimate Strain	0.679	0.941

Table S2: ANCOVA With Interaction Factors for Elastic Modulus, Yield Stress, and Ultimate Strength. Infill density and shore hardness were the strongest predictors ($F \approx$ 8E3–1.5E4), followed by pattern and anchor presence ($F \approx$ 2E2–8E2). Most two- and three-way interactions were also significant ($p < 0.001$), while higher-order terms showed smaller effects.

Mechanical Response:	Elastic Modulus		Yield Stress		Ultimate Tensile Strength	
Factor / Interaction	**F**	**p-value**	**F**	**p-value**	**F**	**p-value**
Pattern	431.62	1.55E-55	289.78	1.15E-46	168.68	1.37E-35

Anchor Presence	445.60	3.25E-42	264.49	3.97E-32	677.33	3.54E-51
Shore Hardness	1.53E4	2.01E-128	8.03E3	9.11E-112	9.21E3	2.67E-115
Infill Density	1.46E4	3.01E-160	8.47E3	4.30E-146	9.94E3	3.15E-150
Pattern × Anchor	62.71	2.28E-19	30.45	2.02E-11	57.23	3.52E-18
Pattern × Shore Hardness	154.91	5.67E-34	99.92	2.85E-26	188.13	1.02E-37
Anchor × Shore Hardness	16.23	9.89E-05	20.28	1.56E-05	145.04	2.20E-22
Infill Density × Anchor	3.98	4.57E-03	3.79	6.11E-03	3.94	4.82E-03
Infill Density × Shore Hardness	1.47E3	5.67E-101	8.06E2	1.18E-85	7.14E2	1.26E-82
Pattern × Infill Density	306.53	4.68E-76	177.51	9.86E-63	134.57	3.45E-56
Pattern × Infill Density × Anchor	1.82	8.06E-02	1.30	2.50E-01	5.41	7.92E-06
Pattern × Infill Density × Shore Hardness	50.65	6.26E-35	28.55	1.92E-24	47.56	1.09E-33
Infill Density × Anchor × Shore Hardness	21.00	3.82E-13	11.01	1.22E-07	4.08	3.88E-03
Pattern × Anchor × Shore Hardness	37.35	2.45E-13	19.94	3.35E-08	24.49	1.20E-09
Pattern × Infill Density × Anchor × Shore Hardness	10.02	1.28E-10	6.11	1.36E-06	13.23	1.45E-13

Table S3: Adaptive Cubic TPU 85A.

	Infill	Elastic Mod	Yield σ	Yield Strain	Ultimate σ	Ultimate Strain
Anchor	20%	0.70 ± 0.09	0.14 ± 0.07	0.296 ± 0.121	1.30 ± 0.03	7.92 ± 0.02
Anchor	40%	1.01 ± 0.06	0.15 ± 0.06	0.188 ± 0.075	2.02 ± 0.02	7.54 ± 0.37
Anchor	60%	2.44 ± 0.27	0.43 ± 0.12	0.223 ± 0.082	3.79 ± 0.07	7.89 ± 0.03
Anchor	80%	3.66 ± 0.17	0.70 ± 0.04	0.220 ± 0.011	5.06 ± 0.06	7.87 ± 0.02
Anchor	99.9%	7.95 ± 0.18	1.52 ± 0.07	0.248 ± 0.004	7.99 ± 0.17	7.90 ± 0.01
No Anchor	20%	0.52 ± 0.07	0.08 ± 0.04	0.185 ± 0.062	0.98 ± 0.04	7.70 ± 0.33
No Anchor	40%	1.06 ± 0.13	0.17 ± 0.04	0.192 ± 0.015	1.86 ± 0.07	7.69 ± 0.18
No Anchor	60%	2.20 ± 0.01	0.43 ± 0.07	0.254 ± 0.045	3.62 ± 0.02	7.91 ± 0.02
No Anchor	80%	3.48 ± 0.08	0.64 ± 0.03	0.266 ± 0.013	4.78 ± 0.08	7.92 ± 0.02
No Anchor	99.9%	7.85 ± 0.09	1.48 ± 0.03	0.280 ± 0.007	7.93 ± 0.04	7.93 ± 0.02

Table S4: Adaptive Cubic TPU 95A.

	Infill	Elastic Mod	Yield σ	Yield Strain	Ultimate σ	Ultimate Strain
Anchor	20%	2.34 ± 0.10	0.40 ± 0.06	0.214 ± 0.022	2.63 ± 0.07	5.23 ± 0.16
Anchor	40%	3.65 ± 0.09	0.66 ± 0.03	0.235 ± 0.013	3.80 ± 0.17	4.52 ± 0.16
Anchor	60%	8.00 ± 0.20	1.50 ± 0.02	0.232 ± 0.006	9.03 ± 0.15	6.08 ± 0.20
Anchor	80%	11.54 ± 0.04	2.16 ± 0.05	0.251 ± 0.011	11.59 ± 0.09	5.89 ± 0.11
Anchor	99.9%	16.34 ± 0.53	3.02 ± 0.10	0.243 ± 0.012	13.51 ± 0.21	5.92 ± 0.17
No Anchor	20%	2.05 ± 0.07	0.32 ± 0.06	0.210 ± 0.028	1.35 ± 0.06	3.49 ± 0.41
No Anchor	40%	3.19 ± 0.11	0.59 ± 0.03	0.237 ± 0.015	3.46 ± 0.12	4.79 ± 0.41
No Anchor	60%	7.93 ± 0.22	1.41 ± 0.02	0.223 ± 0.015	8.31 ± 0.18	6.35 ± 0.18
No Anchor	80%	11.81 ± 0.29	2.16 ± 0.05	0.263 ± 0.019	10.80 ± 0.14	6.30 ± 0.14
No Anchor	99.9%	15.32 ± 0.27	2.83 ± 0.09	0.258 ± 0.030	13.13 ± 0.25	6.45 ± 0.06

Table S5: 3D Honeycomb TPU 85A.

	Infill	Elastic Mod	Yield σ	Yield Strain	Ultimate σ	Ultimate Strain
Anchor	20%	0.59 ± 0.11	0.09 ± 0.04	0.228 ± 0.030	0.90 ± 0.13	3.03 ± 0.86
Anchor	40%	1.61 ± 0.21	0.27 ± 0.04	0.237 ± 0.024	2.38 ± 0.29	4.76 ± 0.94
Anchor	60%	3.54 ± 0.25	0.69 ± 0.05	0.263 ± 0.006	3.62 ± 0.03	5.40 ± 0.64
Anchor	80%	7.81 ± 0.26	1.50 ± 0.08	0.271 ± 0.029	6.24 ± 0.52	5.62 ± 1.31
Anchor	99.9%	12.70 ± 0.19	2.49 ± 0.06	0.277 ± 0.005	10.69 ± 0.09	7.95 ± 0.05
No Anchor	20%	0.28 ± 0.07	0.07 ± 0.01	0.198 ± 0.077	0.26 ± 0.07	2.10 ± 0.39
No Anchor	40%	0.64 ± 0.08	0.17 ± 0.10	0.300 ± 0.112	1.70 ± 0.03	6.21 ± 0.28
No Anchor	60%	1.55 ± 0.08	0.45 ± 0.20	0.337 ± 0.144	2.88 ± 0.30	6.05 ± 1.83
No Anchor	80%	4.58 ± 0.12	0.92 ± 0.02	0.278 ± 0.016	4.76 ± 0.61	6.05 ± 1.00
No Anchor	99.9%	11.86 ± 0.40	2.30 ± 0.06	0.294 ± 0.012	10.05 ± 0.18	7.94 ± 0.01

Table S6: 3D Honeycomb TPU 95A.

	Infill	Elastic Mod	Yield σ	Yield Strain	Ultimate σ	Ultimate Strain
Anchor	20%	1.57 ± 0.14	0.24 ± 0.03	0.203 ± 0.042	1.58 ± 0.05	2.79 ± 0.26
Anchor	40%	3.47 ± 0.14	0.66 ± 0.04	0.232 ± 0.005	4.69 ± 0.14	5.52 ± 0.25
Anchor	60%	5.93 ± 0.05	1.09 ± 0.05	0.237 ± 0.012	6.94 ± 0.19	6.59 ± 0.58

Anchor	80%	11.83 ± 0.14	2.27 ± 0.05	0.245 ± 0.014	9.05 ± 0.11	4.39 ± 0.33
Anchor	99.9%	23.90 ± 0.39	4.49 ± 0.07	0.266 ± 0.013	16.83 ± 0.27	6.64 ± 0.19
No Anchor	20%	0.49 ± 0.14	0.09 ± 0.05	0.219 ± 0.074	0.52 ± 0.03	1.41 ± 0.22
No Anchor	40%	2.26 ± 0.11	0.38 ± 0.05	0.218 ± 0.010	3.35 ± 0.12	4.83 ± 0.26
No Anchor	60%	5.07 ± 0.21	1.02 ± 0.10	0.248 ± 0.021	5.19 ± 0.16	3.86 ± 0.90
No Anchor	80%	12.22 ± 0.55	2.31 ± 0.13	0.265 ± 0.002	8.20 ± 0.77	3.45 ± 0.69
No Anchor	99.9%	21.64 ± 0.40	4.06 ± 0.06	0.267 ± 0.005	14.64 ± 0.10	5.30 ± 0.29

Table S7: Gyroid Flat TPU 85A.

	Infill	Elastic Mod	Yield σ	Yield Strain	Ultimate σ	Ultimate Strain
Anchor	20%	0.79 ± 0.08	0.15 ± 0.04	0.229 ± 0.003	1.17 ± 0.20	5.14 ± 1.13
Anchor	40%	1.54 ± 0.22	0.23 ± 0.08	0.207 ± 0.065	2.68 ± 0.06	7.32 ± 0.30
Anchor	60%	3.08 ± 0.24	0.62 ± 0.02	0.276 ± 0.031	3.72 ± 0.19	6.23 ± 0.25
Anchor	80%	5.31 ± 0.14	0.97 ± 0.02	0.279 ± 0.023	5.77 ± 0.11	7.94 ± 0.03
Anchor	99.9%	10.97 ± 0.56	2.14 ± 0.12	0.258 ± 0.006	9.34 ± 0.83	6.74 ± 0.74
No Anchor	20%	0.31 ± 0.04	0.03 ± 0.03	0.205 ± 0.112	0.43 ± 0.07	4.19 ± 0.26
No Anchor	40%	1.05 ± 0.06	0.24 ± 0.07	0.272 ± 0.101	1.94 ± 0.14	6.06 ± 0.51
No Anchor	60%	2.62 ± 0.06	0.42 ± 0.11	0.245 ± 0.064	3.29 ± 0.28	6.19 ± 0.61
No Anchor	80%	4.71 ± 0.23	0.90 ± 0.08	0.269 ± 0.026	5.55 ± 0.34	7.19 ± 0.68
No Anchor	99.9%	10.24 ± 0.72	1.95 ± 0.12	0.280 ± 0.005	8.52 ± 0.43	6.71 ± 0.27

Table S8: Gyroid Flat TPU 95A.

	Infill	Elastic Mod	Yield σ	Yield Strain	Ultimate σ	Ultimate Strain
Anchor	20%	2.52 ± 0.12	0.43 ± 0.09	0.239 ± 0.037	2.46 ± 0.02	3.52 ± 0.07
Anchor	40%	5.69 ± 0.41	1.07 ± 0.07	0.256 ± 0.012	5.91 ± 0.48	4.78 ± 0.50
Anchor	60%	9.35 ± 0.44	1.76 ± 0.04	0.261 ± 0.017	9.30 ± 0.35	5.34 ± 0.23
Anchor	80%	15.48 ± 0.09	2.91 ± 0.02	0.266 ± 0.003	14.68 ± 0.06	6.81 ± 0.06
Anchor	99.9%	22.54 ± 0.87	4.25 ± 0.05	0.284 ± 0.014	17.51 ± 0.25	7.18 ± 0.27
No Anchor	20%	0.89 ± 0.13	0.12 ± 0.03	0.165 ± 0.024	1.19 ± 0.05	3.37 ± 0.20
No Anchor	40%	3.58 ± 0.08	0.58 ± 0.09	0.230 ± 0.044	3.51 ± 0.15	3.98 ± 0.27

No Anchor	60%	7.84 ± 0.46	1.45 ± 0.09	0.246 ± 0.009	6.02 ± 0.46	3.66 ± 0.48
No Anchor	80%	13.62 ± 0.53	2.54 ± 0.09	0.270 ± 0.010	11.09 ± 0.26	5.20 ± 0.37
No Anchor	99.9%	20.40 ± 0.42	3.82 ± 0.07	0.280 ± 0.009	16.60 ± 0.43	7.12 ± 0.24

Table S9: Rectilinear TPU 85A.

	Infill	Elastic Mod	Yield σ	Yield Strain	Ultimate σ	Ultimate Strain
Anchor	99.9%	13.59 ± 0.12	2.64 ± 0.05	0.274 ± 0.008	11.75 ± 0.12	7.92 ± 0.01
No Anchor	99.9%	12.65 ± 0.20	2.47 ± 0.05	0.283 ± 0.021	11.55 ± 0.19	7.94 ± 0.02

Table S10: Rectilinear TPU 95A.

	Infill	Elastic Mod	Yield σ	Yield Strain	Ultimate σ	Ultimate Strain
Anchor	99.9%	24.80 ± 0.70	4.65 ± 0.16	0.243 ± 0.006	18.50 ± 0.05	6.13 ± 0.17
No Anchor	99.9%	24.11 ± 0.15	4.49 ± 0.10	0.255 ± 0.006	18.78 ± 0.33	6.53 ± 0.22

Table S11: Mechanical Properties of fresh human Achilles Tendon by Louis-Ugbo et al. [27].

Specimen	Gender	Age (years)	Side	Stiffness (N/mm)	UTS (N)	Cross-sectional area (mm^2)	Modulus of elasticity (N/mm^2 or MPa)
1	M	59	R	67,219	1,217	117.6	572
2	M	59	L	46,435	1,215	114.4	406
3	M	77	R	72,917	1,896	109	669
4	M	77	L	43,750	1,573	102.5	427
5	M	86	R	93,750	1,653	81.25	1,154
6	M	86	L	54,167	1,350	75.35	719
7	M	92	R	25,000	853	123.2	203
8	M	92	L	18,750	715	117.6	159
9	M	93	R	45,000	1,001	120	375
10	M	93	L	87,500	1,965	123	711
11	F	68	R	50,000	857	142.1	352
12	F	68	L	50,000	828	100.5	498
13	F	83	R	58,333	1,402	115.5	505

14	F	83	L	80,000	1,750	92.61	864
15	F	84	R	25,000	350	74.67	335
16	F	85	L	40,000	1,038	60.99	656
17	F	87	R	32,000	430	114.7	279
18	F	87	L	68,900	360	94.54	729
19	M	71	R	66,000	1,730	110.2	599
20	M	71	L	105,000	1,600	108	972
Average		80		56,486	1,189	105	559

Table S12: Quantitative Comparison: Best TPU Sample vs. Human Achilles Tendon by Louis-Ugbo et al. [27].

	TPU (Rectilinear, 95A, Anchor, 100% Infill Density)	Human Achilles Tendon	% Mismatch
Elastic Moduli (MPa)	24.8	1,189	97.9%
Ultimate Tensile Strength (MPa)	18.5	559	96.7%
